# Effectiveness of oral famotidine in reducing the hematologic complications of radiotherapy in patients with esophageal and cardia cancers: a randomized controlled trial

**DOI:** 10.1186/s13014-023-02281-w

**Published:** 2023-05-20

**Authors:** Mina Rostami, Omid Yelghi, Zhaleh Karimi Moghaddam, Alireza Zeraatchi, Hamed Rezaeejam, Alireza Sadeghi

**Affiliations:** 1 Social Determinants of Health Research Center, Zanjan University of Medical Sciences, Zanjan, Iran; 2 Department of Radiation Oncology, School of Medicine, Vali-e-Asr Hospital, Zanjan University of Medical Sciences, Zanjan, Iran; 3 Department of Emergency Medicine, School of Medicine, Valiasr-e-Asr Hospital, Ayatollah Mousavi Hospital, Zanjan University of Medical Sciences, Zanjan, Iran; 4 Department of Radiology Technology, School of Paramedical Sciences, Zanjan University of Medical Sciences, Zanjan, Iran; 5 Department of Internal Medicine, School of Medicine, Vali-e-Asr Hospital, Zanjan University of Medical Sciences, Zanjan, Iran

**Keywords:** Famotidine, Radioprotective agents, Esophageal neoplasms, Cardia, Thrombocytopenia

## Abstract

**Background:**

Chemoradiotherapy complications has always been of great concern to both clinicians and patients during the course of treatment. The purpose of the present study was to examine the effectiveness of oral famotidine on the reduction of hematologic complications of patients with esophageal and gastric cardia cancers undergoing radiotherapy.

**Methods:**

A single-blind controlled trial was conducted on 60 patients with esophageal and cardia cancers, who were undergoing chemoradiotherapy. Patients were randomly assigned to 2 groups with 30 patients to receive either 40 mg of oral famotidine (daily and 4 h before each session) or placebo. Complete blood count with differential, platelet counts, and hemoglobin levels were obtained weekly during treatment. The main outcome variables were lymphocytopenia, granulocytopenia, thrombocytopenia, and anemia.

**Results:**

The findings indicated a significant effect of famotidine on reduction of thrombocytopenia among intervention group compared to control group (*P* < 0.0001). Even so, the effect of intervention was not significant for other outcome variables (All, *P* ≥ 0.05). The lymphocyte (*P* = 0.007) and platelet (*P* = 0.004) counts were also significantly greater in famotidine group in comparison with placebo group at the end of the study.

**Conclusion:**

As evidenced by the findings of the current study, famotidine might be recommended as an effective radioprotective agent among patients with esophageal and gastric cardia cancers to prevent Leukocyte and platelet reduction to some extent.

*Trial registration* This study was prospectively registered at irct.ir (Iranian Registry of Clinical Trials) with the code IRCT20170728035349N1, 2020-08-19.

## Background

According to recent estimates, by 2025 the prevalence of cancer is expected to soar by 45% in developed countries. Cancer is the second cause of death worldwide following cardiovascular diseases. More than one million cases of gastric cardia and 450,000 cases of esophageal cancers are being diagnosed annually, which are the seventh and eighth most prevalent cancers, respectively [1–4]. In Iran, according to the recent predictions, the incidence of cancer is expected to increase by 42.6% until 2025. In other words, the number of new cancer cases is predicted to rise from 112,000 cases registered in 2016 to an estimated 160,000 cases in 2025. Meanwhile, in 2016, breast, colorectal and stomach cancers were the most commonly diagnosed cancers, being predicted to remain the top three cancer types in 2025 [5].

Chemo-radiotherapy with or without surgery is an approved and favored treatment strategy in gastric cardia and esophageal cancers. Esophagitis and aggravation of transient dysphagia is witnessed in approximately 75% of patients who undergo chemoradiotherapy. Besides, they may experience early acute complications such as dermatitis, fatigue, weight loss, nausea and vomiting, as well as hematological complications like lymphopenia, granulocytopenia, thrombocytopenia and finally anemia [6].

Irradiation-induced dose-dependent decrease occurs in all hematopoietic cell lineage. Reduction in the number of lymphocytes, granulocytes, and red blood cells occurs in hours, days, and weeks after the initiation of treatment, respectively [7]. Through studying murine models, the effects of irradiation on hematopoiesis have thoroughly been investigated, namely it causes oxidative damage, negatively affects DNA damage repair and cell proliferation, and promotes cell senescence [8]. Among them, leukocytes, especially lymphocytes, are the most radio-sensitive cells [7, 8]. Moreover, several studies have demonstrated that the drop in the number of lymphocytes remains for years following the radiotherapy [9–11], which has a significant impact on reducing the prognosis of patients with cancer [12–14].

The importance of the adverse consequences of radiotherapy particularly the hematological complications becomes more prominent with acknowledging the fact that radiotherapy is widely used all over the world for either palliative or curative purposes. Of nearly 10.9 million people who are diagnosed with cancer annually, radiation therapy is given to about 60%, among them 40% are meant for curative treatment [15].

Effective and timely management of radiotherapy complications should be taken seriously. For radiation oncologists, identifying an efficient and non-toxic radio-protective agent has always been essential. Radio-protective agents are utilized in order to prevent or reduce cellular damage due to ionizing radiation. They are often antioxidants that must be administered concomitantly or prior to the treatment [15].

Sulfur compounds such as amifostine are the first compounds that have been evaluated as radio-protective agents. Evidence suggests that amifostine is a potent and systemically effective radioprotector when it is administered in high doses. However, it shows toxicity at the high doses required for radioprotection that pertains to survival benefits. Its severe side effects (e.g. nausea, vomiting, and hypotension), along with the non-oral route, and the need for blood pressure monitoring during injection have hindered its use. Therefore, there is still a considerable need for an effective agent with minor side effects [16].

It has been shown that H2 receptor antagonists such as cimetidine and famotidine have radioprotective effects. Famotidine has been known as a strong agent to reduce radiation-induced apoptosis, as well as lipid peroxidation, DNA damage, chromosomal aberrations, micronuclei formation, and lethality [17].

Existing evidence confirms that famotidine has been a promising agent in protecting leukocytes from radiation-induced apoptosis [18]. It has also been demonstrated that famotidine can lead to a significant drop in radiation-induced lymphocytopenia [19]. Yet, there is scarce evidence supporting the use of famotidine as a redioprotective drug. Studies especially clinical trials in the field of radioprotective agents have been conducted inadequately. Addressing this issue can substantially contribute to enhancing the tolerance of radiation therapy, increasing life expectancy, and improving the prognosis of cancer. The present study is designed to examine famotidine as a radioprotective drug in reducing the hematological complications of radiation therapy among a population of patients with cardia and esophageal cancers.

## Methods and materials

### Trial design and participants

This was a single center, parallel designed (1:1), single-blind randomized placebo-controlled trial performed on patients with esophageal and gastric cardia cancers who were referred to the radiation oncology clinic of the Vali-E-asr Hospital, Zanjan, Iran, from September 2020 to December 2021.

### Ethical considerations

The present study was approved by the Ethics Committee of Zanjan University of Medical Sciences [IR.ZUMS.REC.1399.158]. Written informed consent was obtained from all participant. Patients entered the study with their full awareness and willingness. Patients could withdraw from the study at any time. The medicine used in the present study has potentially no serious side effects. No prescription charges were obtained from patients.

### Eligibility criteria

The inclusion criteria were considered the patients aged ≥ 18 years with non-metastatic esophageal and cardia cancers who were candidates for chemo-radiotherapy and did not receive Proton pump inhibitors (PPIs) and other H2-blockers.

Death during the study, being unable to continue treatment due to the severity of treatment complications, having renal impairment, being hypersensitive to H2-blockers, using any other H2-blockers (e.g. cimetidine) simultaneously and need to use PPI family medicines during the treatment process, caused an exclusion from the study.

### Interventions

This trial was designed to determine the effectiveness of oral famotidine on reducing the acute hematological complications of radiotherapy among patients with esophageal and cardia cancers. All patients received a fixed therapeutic dose of external beam radiation therapy. 15MV high-energy photon from linear accelerator (Siemens Primus, Siemens Medical Systems, Concord, CA, USA) was used for treatment. Patients were treated with 3 fields per day with a dose rate of 180 cGy/day, five days/week. Planning target volume (PTV) was 5 mm from clinical target volume (CTV). Dose-volume histogram (DVH) was considered mean dose of 30 Gy and mean dose of 15 Gy for liver and spleen, respectively. Moreover, patients received the chemotherapy regimen of paclitaxel 80 mg/m^2^ and carboplatin 2 mg/m^2^ weakly simultaneous with radiotherapy. The intervention group received oral famotidine 40 mg tablets (®FAMOTED, Tehrandarou Co, 2020, Tehran, Iran), administered once a day 4 h before each radiotherapy session. Patients received a total of 20 sessions of radiotherapy 5 consecutive days a week and followed up for 4 weeks. All patients were fasting from 2 h before the treatment and each treatment session began from 11 a.m. onwards. The control group received placebo. To ensure adherence to the treatment, the participants were instructed before the beginning of the study and provided with written instructions. They also were followed up on taking the medications properly before initiation of each session.

### Outcomes

Acute hematological complications of radiotherapy including lymphocytopenia, granulocytopenia, thrombocytopenia, and anemia were the outcome variables of the study that were assessed once at baseline and then weekly at the end of each week using complete blood count (CBC) with differential at the laboratory of Vali-e-Asr hospital by blinded laboratory staff using KX-21N hematology analyzer (Sysmex Corporation, Kobe, Hyogo, Japan). The results of lab tests were then collected and evaluated by an assessor blinded to random allocation of study groups. The variables of age, gender, the week in which lab tests were done and the type of cancer were also collected using a checklist.

### Sample size

The sample size was determined based on the study of Razzaghdoust et al. [19], using OpenEpi [20] web-based tool version 3.01. As α = 0.05, the statistical power = 80%, percent of exposed with outcome = 20%, and percent of unexposed with outcome = 55%, the sample size was calculated to be 30 for each group.

### Randomization and blinding

Participants, laboratory staff, data collectors, outcome assessors were blinded by not informing them of the group allocations. Data analysts were blinded by providing them with a blinded version of the data. The principal Investigator was aware of treatment assignments. Random sequence of participants was made using Blocked randomization (blocks of 4) by Microsoft Excel, using the RAND function. Sequentially numbered, opaque, sealed envelopes were used to conceal the allocation sequence from the researchers who were enrolling and assessing the participants. Outcome assessment was done by the same assessor for two groups.

### Statistical analyses

**Fig. 1:**
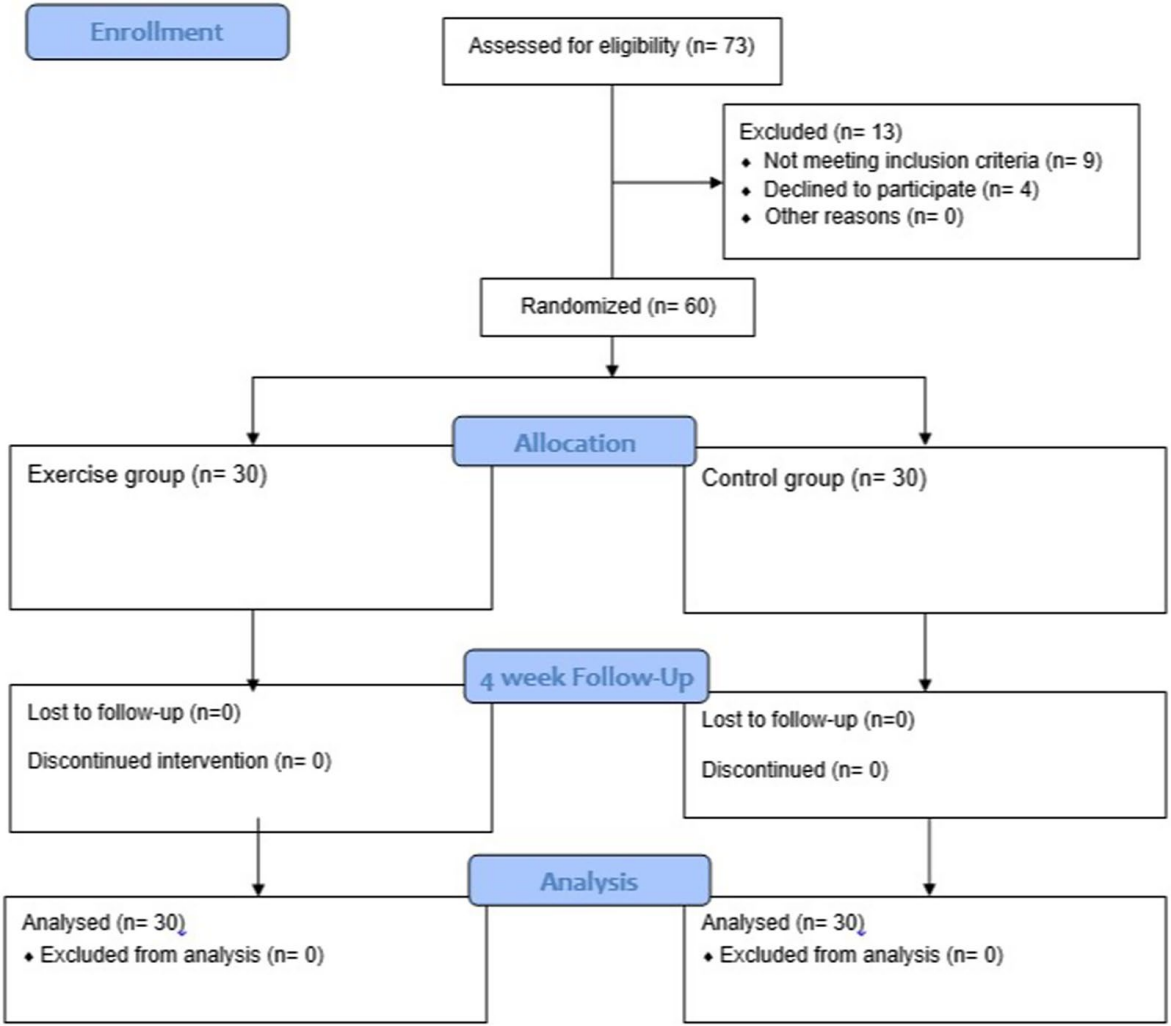
The CONSORT flow diagram

Shapiro–Wilk test was used to test the normality of the numeric data. Mean ± standard deviation (SD), median (IQR) and frequency (%) were used to report descriptive statistics, as applicable. Between-group comparisons were examined using Mann–Whitney U test and independent samples t-test with mean difference (MD) and 95% confidence interval (95%CI). Within-group comparisons were investigated through Friedman test with a post-hoc analysis using Wilcoxon signed-rank test with Bonferroni correction. *P*-value less than 0.05 was considered statistically significant. Statistical analysis was performed using IBM SPSS Statistics software version 26 (IBM SPSS Statistics, Armonk, NY, USA). This study was conducted in line with the Consolidated Standards of Reporting Trial (CONSORT) (Fig. 1).

## Results

### Basic characteristics of the participants

A total of 60 patients (32 male, 28 female) with stage I–III esophageal cancer including squamous cell carcinoma and adenocarcinoma, in addition to stage I–III Siewert type I esophagogastric junction (EGJ) and gastric cardia adenocarcinomas were included in the study.

With regard to the gender ratio of the participants, in both groups there was 16 (53.3%) males and 14 (46.7%) females.

Mean ± SD age of total participants was 66.55 ± 11.57. Mean age of participants in control group was 67.17 ± 12.73 and in intervention group was 65.93 ± 10.46. We found that there was no significant difference between the two groups in terms of age (t(58) = 0.410, *P* = 0.683).

### Outcome variables

**Fig. 2:**
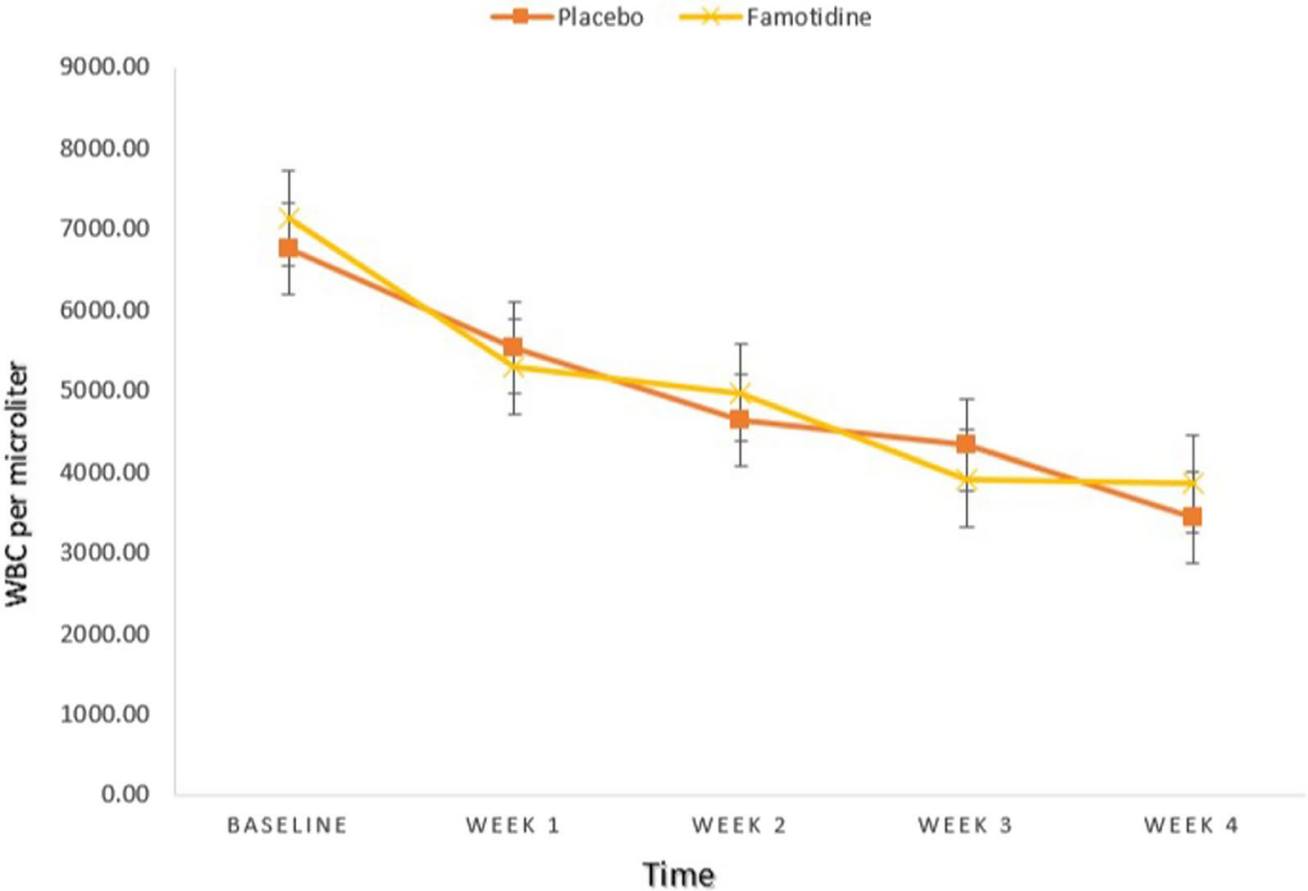
Between-group comparisons of WBC

**Fig. 3:**
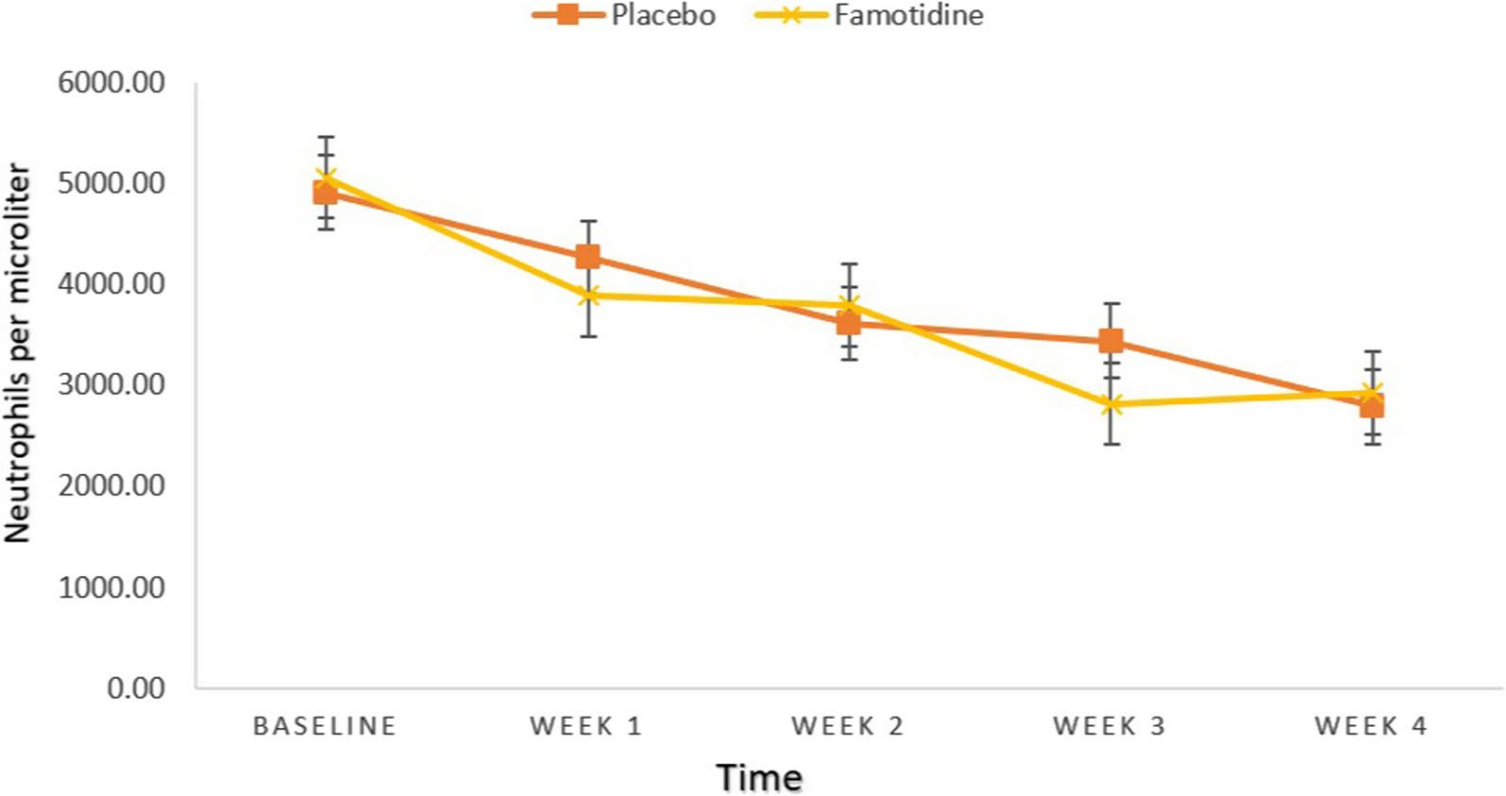
Between-group comparisons of Neutrophil

An independent samples t-test revealed that there was no significant difference between the two groups considering white blood cells (WBC) and Neutrophil counts at baseline and week 1, 2, 3, 4 (All, *P*-value ≥ 0.05) (Tables 1, 2) (Figs. 2, 3).

**Table 1:** Between-group comparisons of WBC (Per microliter)

	Placebo group		Famotidine group		MD (95%CI)	t	d	*P*-value*
	Mean	SD	Mean	SD				
WBC 0	6761.00	1765.66	7125.00	2099.78	− 364.00 (− 1366.63, 638.63)	− 0.727	0.188	0.470
WBC 1	5532.00	1718.40	5288.00	1501.55	244.00 (− 589.99, 1077.99)	0.586	− 0.151	0.560
WBC 2	4619.33	2165.76	4966.67	2116.49	− 347.33 (− 1454.02, 759.36)	− 0.628	0.162	0.532
WBC 3	4328.00	2656.56	3905.67	1766.63	422.33 (− 743.61, 1588.28)	0.725	− 0.187	0.471
WBC 4	3439.33	1438.36	3849.00	1844.35	− 409.66 (− 1264.45, 445.11)	− 0.959	0.248	0.341

*MD* Mean Difference, *WBC* White Blood Cell, *d* effect size (Cohen’s d)

^*^P < 0.05, All *P*-values are obtained from independent samples t-test

**Table 2:** Between-group comparisons of Neutrophil (Per microliter)

	Placebo group		Famotidine group		MD (95%CI)	t	d	*P*-value*
	Mean	SD	Mean	SD				
Neutrophil 0	4915.67	1711.11	5059.67	1879.03	− 144.00 (− 1072.78, 784.78)	− 0.310	0.080	0.757
Neutrophil 1	4270.63	1586.87	3889.67	1155.37	380.96 (− 336.40, 1098.33)	1.063	− 0.274	0.292
Neutrophil 2	3613.33	2311.40	3795.90	1763.01	− 182.56 (− 1244.97, 879.84)	− 0.344	0.089	0.732
Neutrophil 3	3442.00	2150.37	2819.93	1375.48	622.06 (− 310.83, 1554.96)	1.335	− 0.345	0.187
Neutrophil 4	2790.93	1382.96	2926.97	1533.45	− 136.03 (− 890.70, 618.63)	− 0.361	0.093	0.720

*MD* Mean Difference, *d* effect size (Cohen’s d)

^*^P < 0.05, All *P*-values are obtained from independent samples t-test

**Fig. 4:**
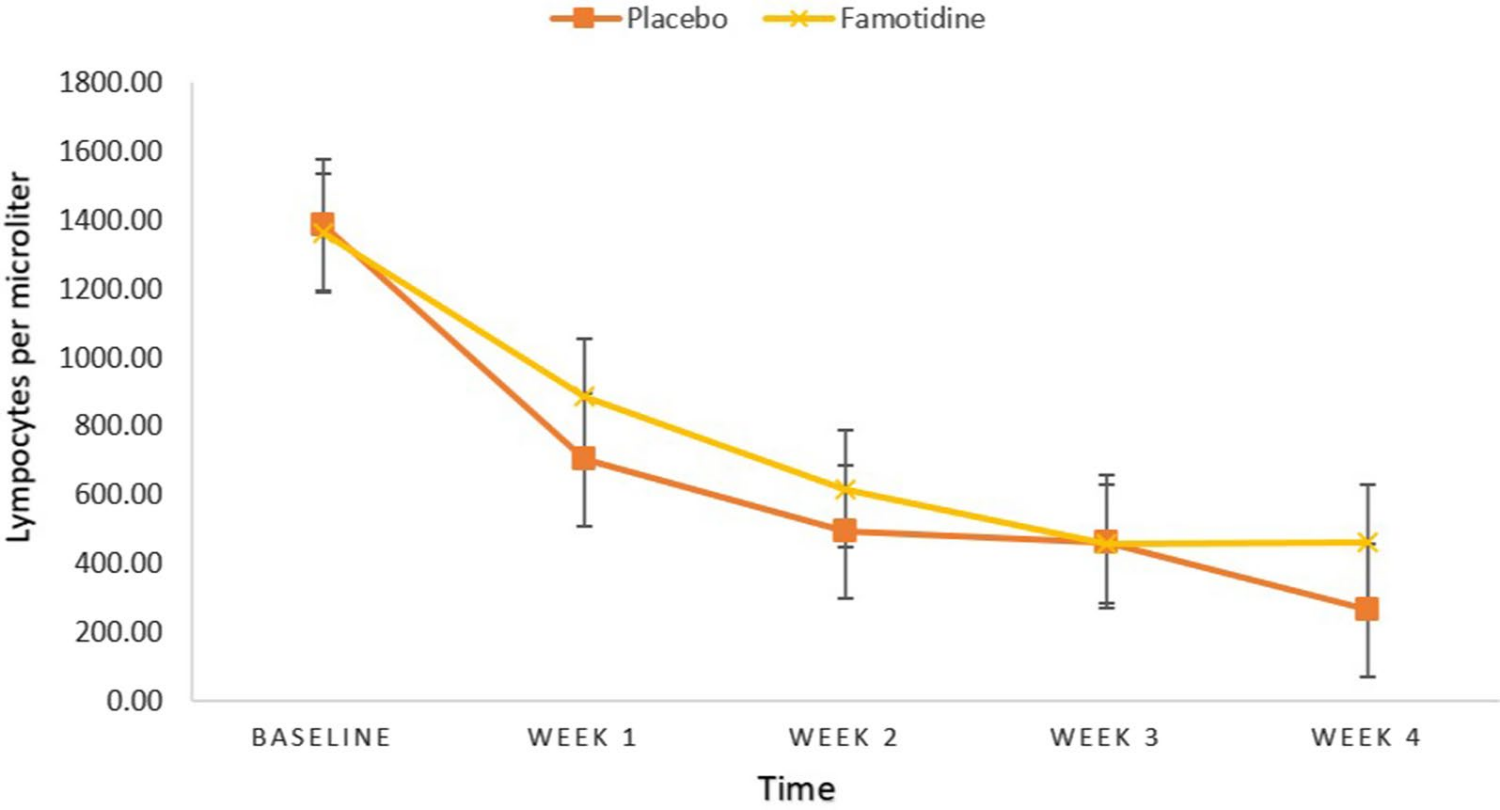
Between-group comparisons of Lymphocyte

A Mann–Whitney U test demonstrated that at week 4 Lymphocyte count was greater for intervention group (Median (Mdn) = 460.00) than for control group (Mdn = 265.50) with a large effect size (d = 0.736), U = 269.00, *P*-value = 0.007. Even so, there was no significant difference between the two groups at baseline, week 1, 2, and 3 (All, *P*-value ≥ 0.05) (Table 3, Fig. 4).

**Table 3:** Between-group comparisons of Lymphocyte (Per microliter)

	Placebo group		Famotidine group		Mann–Whitney U	Z	d	*P*-value*
	Mean rank	Sum of ranks	Mean rank	Sum of ranks				
Lymphocyte 0	30.00	900.00	31.00	930.00	435.00	− 0.222	0.057	0.824
Lymphocyte 1	27.02	810.50	33.98	1019.50	345.50	− 1.545	0.407	0.122
Lymphocyte 2	26.37	791.00	34.63	1039.00	326.00	− 1.833	0.487	0.067
Lymphocyte 3	27.82	834.50	33.18	995.50	369.50	− 1.190	0.311	0.234
Lymphocyte 4	24.47	734.00	36.53	1096.00	269.00	− 2.676	0.736	**0.007**

^*^P < 0.05, All *P*-values are obtained from Mann Whitney U Test. d, effect size (Cohen’s d)

Significant *p*-values are shown in bold

**Fig. 5:**
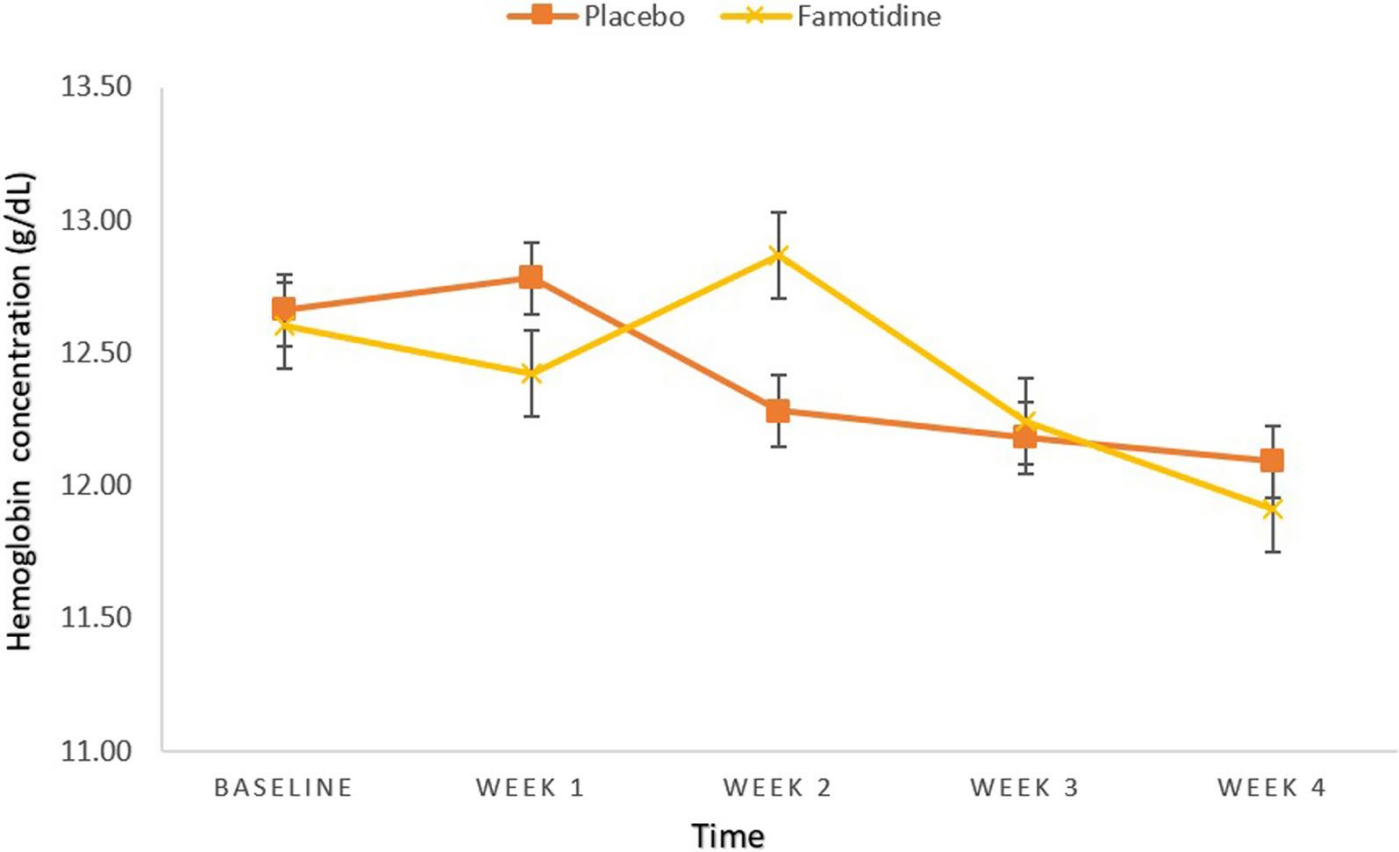
Between-group comparisons of Hemoglobin concentration

Hemoglobin concentration (g/dL) also showed no significant difference between two groups at baseline and week 1, 2, 3, 4 (All, *P*-value ≥ 0.05) (Table 4, Fig. 5).

**Table 4:** Between-group comparisons of Hb (g/dL)

	Placebo group		Famotidine group		MD (95%CI)	t	d	*P*-value*
	Mean	SD	Mean	SD				
Hb 0	12.66	1.75	12.60	1.35	0.06 (− 0.74, 0.86)	0.148	− 0.038	0.883
Hb 1	12.78	1.59	12.42	1.57	0.35 (− 0.46, 1.17)	0.871	− 0.228	0.387
Hb 2	12.28	1.55	12.87	2.09	− 0.58 (− 1.54, 0.36)	− 1.231	0.321	0.223
Hb 3	12.18	1.63	12.24	1.84	− 0.06 (− 0.96, 0.83)	− 0.148	0.035	0.883
Hb 4	12.09	1.43	11.91	1.70	0.17 (− 0.63, 0.99)	0.434	− 0.115	0.666

*MD* Mean Difference, *Hb* Hemoglobin, *d* effect size (Cohen’s d)

^*^P < 0.05, All *P*-values are obtained from independent samples t-test

**Fig. 6:**
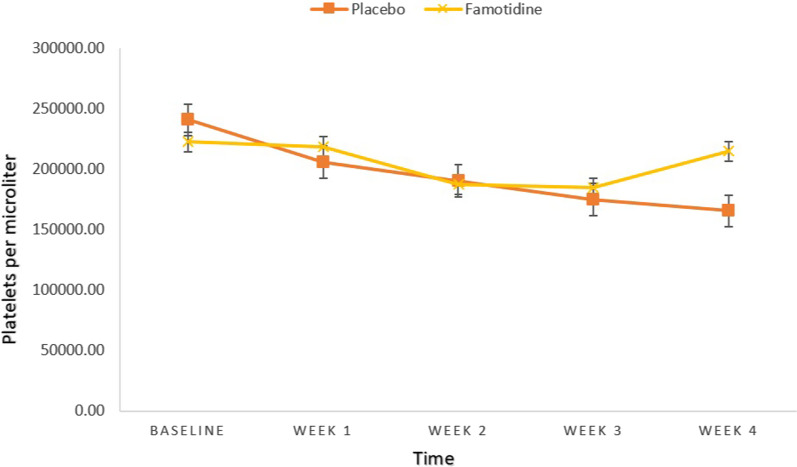
Between-group comparisons of Platelets

At week 4 patients in intervention group found to have higher levels of Platelet count than patients in control group (214,966.67 Vs. 165,633.33, t(58) = − 3.024, *P*-value = 0.004), with a large effect size (d = 0.781). However, at baseline, week 1, 2 and 3 there was no significant difference between two groups (All, *P*-value ≥ 0.05) (Table 5, Fig. 6).

**Table 5:** Between-group comparisons of Plt (Per microliter)

	Placebo group		Famotidine group		MD (95%CI)	t	d	*P*-value*
	Mean	SD	Mean	SD				
Plt 0	240,800.00	70,968.86	222,733.33	48,652.84	18,066.66 (− 13,379.37, 49,512.70)	1.150	− 1.053	0.255
Plt 1	205,833.33	59,125.281	218,766.67	46,818.56	− 12,933.33 (− 40,495.50, 14,628.83)	− 0.939	0.243	0.351
Plt 2	190,500.00	56,931.14	187,566.67	54,024.05	2933.33 (− 25,749.61, 31,616.28)	0.205	− 0.053	0.839
Plt 3	174,966.67	62,561.13	184,766.67	45,832.29	− 9800.00 (− 38,142.73, 18,542.73)	− 0.692	0.179	0.492
Plt 4	165,633.33	64,058.42	214,966.67	62,277.73	− 49,333.33 (− 81,984.44, − 16,682.22)	− 3.024	0.781	**0.004**

*MD* Mean Difference, *Plt* Platelets, *d* effect size (Cohen’s d)

^*^P < 0.05, All *P*-values are obtained from independent samples t-test

Significant *p*-values are shown in bold

Post-intervention changes of outcome variables are shown in Table 6. Between-group analysis of outcome variable changes revealed that there was merely significant differences between two groups in terms of Platelet count changes, so that a Mann–Whitney U test indicated that from baseline to week 1 (Mdn, − 14.12 Vs. − 4.96), week 2 to 3 (Mdn, − 25.33 Vs. − 12.24) as well as week 3 to 4 (Mdn, − 25.33 Vs. − 12.24), the reduction in the Platelet count (thrombocytopenia) was higher for control group compared to intervention group (U = 302.00, *P*-value = 0.029, U = 293.00, *P*-value = 0.020, and U = 179.00, *P* < 0.0001, respectively). There was no other significant differences between two groups in terms of WBC, Neutrophil, Lymphocyte and Hemoglobin changes (Tables 6, 7).

**Table 6:** Post-intervention changes of outcome variables

Outcome changes (%)	Median (IQR)	
	Placebo group	Famotidine group
WBC 0–1	− 22.01 (31.18)	− 24.66 (27.41)
WBC 1–2	− 40.83 (32.84)	− 30.57 (30.58)
WBC 2–3	− 47.06 (37.25)	− 45.44 (43.15)
WBC 3–4	− 50.22 (48.89)	− 46.37 (33.12)
Neutrophil 0–1	− 16.79 (31.68)	− 19.20 (22.39)
Neutrophil 1–2	− 35.20 (45.06)	− 20.62 (41.95)
Neutrophil 2–3	− 34.41 (64.32)	− 42.61 (39.29)
Neutrophil 3–4	− 45.60 (54.89)	− 38.82 (34.53)
Lymphocyte 0–1	− 39.61 (60.55)	− 34.77 (48.51)
Lymphocyte 1–2	− 61.95 (34.07)	− 50.68 (3.08)
Lymphocyte 2–3	− 66.60 (57.49)	− 55.84 (44.24)
Lymphocyte 3–4	− 73.93 (43.11)	− 65.00 (− 4.46)
Hb 0–1	− 2.46 (2.35)	− 1.54 (4.67)
Hb 1–2	− 3.51 (17.54)	2.42 (11.94)
Hb 2–3	− 3.64 (13.85)	− 4.34 (7.67)
Hb 3–4	− 6.10 (13.74)	− 5.65 (9.53)
Plt 0–1	− 14.12 (20.57)	− 4.96 (20.31)
Plt 1–2	− 17.74 (21.52)	− 12.24 (24.43)
Plt 2–3	− 25.33 (23.17)	− 12.24 (24.43)
Plt 3–4	− 31.05 (26.91)	− 2.44 (35.66)

**Table 7:** Between-group comparisons of outcome variable changes

Outcome changes (%)	Placebo group		Famotidine group		Mann–Whitney U	Z	d	*P*-value*
	Mean rank	Sum of ranks	Mean rank	Sum of ranks				
WBC 0–1	29.10	873.00	31.90	957.00	408.00	− 0.621	0.161	0.535
WBC 1–2	33.28	998.50	27.72	831.50	366.50	− 1.235	0.323	0.217
WBC 2–3	30.07	902.00	30.93	928.00	437.00	− 0.192	0.050	0.848
WBC 3–4	31.53	946.00	29.47	884.00	419.00	− 0.458	0.119	0.647
Neutrophil 0–1	29.03	871.00	31.97	959.00	406.00	− 0.651	0.169	0.515
Neutrophil 1–2	33.37	1001.00	27.63	829.00	364.00	− 1.271	0.333	0.204
Neutrophil 2–3	28.63	859.00	32.37	971.00	394.00	− 0.828	0.215	0.408
Neutrophil 3–4	30.83	925.00	30.17	905.00	440.00	− 0.148	0.038	0.882
Lymphocyte 0–1	31.80	954.00	29.20	876.00	411.00	− 0.577	0.149	0.564
Lymphocyte 1–2	33.97	1019.00	27.03	811.00	346.00	− 1.538	0.405	0.124
Lymphocyte 2–3	32.73	982.00	28.27	848.00	383.00	− 0.991	0.258	0.322
Lymphocyte 3–4	34.03	1021.00	26.97	809.00	344.00	− 1.567	0.413	0.117
Hb 0–1	30.58	917.50	30.42	912.50	447.50	− 0.037	0.010	0.971
Hb 1–2	34.10	1023.00	26.90	807.00	342.00	− 1.597	0.421	0.110
Hb 2–3	30.87	926.00	30.13	904.00	439.00	− 0.163	0.042	0.871
Hb 3–4	29.45	883.50	31.55	946.50	418.50	− 0.466	0.120	0.641
Plt 0–1	35.43	1063.00	25.57	767.00	302.00	− 2.188	0.589	**0.029**
Plt 1–2	33.07	992.00	27.93	838.00	373.00	− 1.138	0.297	0.255
Plt 2–3	35.73	1072.00	25.27	758.00	293.00	− 2.321	0.628	**0.020**
Plt 3–4	39.53	1186.00	21.47	644.00	179.00	− 4.007	1.209	** < 0.0001**

^*^P < 0.05, All *P*-values are obtained from Mann Whitney U Test. d, effect size (Cohen’s d)

Significant *P*-values are shown in bold

## Discussion

In the current study we assessed the effectiveness of oral famotidine as a redioprotective agent in reducing hematological complications of radiotherapy among a population of patients with gastric cardia and esophageal cancers. Our findings revealed that there was a significant reduction in thrombocytopenia in the famotidine group compared to placebo group. Moreover, the lymphocyte and platelet counts were also significantly different between famotidine and placebo groups after four weeks.

In a study conducted by Razzaghdoust et al., the effect of famotidine (80 mg/day) as a radioprotective agent on rectal mucosa was evaluated in a group of prostate cancer patients treated with radiotherapy [21]. Findings of the aforementioned study indicated that in comparison with placebo group, patients receiving famotidine demonstrated significantly less grade II rectal toxicity. Additionally, rectal bleeding merely occurred in the placebo group during treatment, and a reduction in the length of rectal toxicity during the radiotherapy course was also evidenced in the famotidine group.

Consistent with the results of our study, it was previously shown that among a population of prostate cancer patients undergoing radiation therapy, famotidine had a significant impact on reducing lymphocytopenia caused by radiation therapy [19].

Also in murine models, the significant role of famotidine as a radioprotector has been observed [17]. Three doses of famotidine (5, 10 and 20 mg/kg) were administered to adult male Naval Medical Research Institute (NMRI) mice intraperitoneally 2 h before irradiation. As evidenced by Zangeneh et al., famotidine at all three doses equally led to a decrease in the number of micronucleated polychromatic erythrocytes (MnPCEs) after treatment using both doses of radiation with same protection factor (~ 2). They inferred that since there was no significant difference between three doses of famotidine in reducing the frequency of MnPCE, it could be concluded that famotidine is likely to act as a potent hydroxyl radical scavenger even at much lower doses than ones used in the study, resulting in anti-clastogenic effects.

In another murine study, radioprotective effect of vitamin C and famotidine has been indicated, as they could significantly decrease cytotoxic effect of radiation on spermatogenesis in mice by probable mechanism of radical scavenging [22]. The radical scavenging mechanism has been suggested in another study as they have asserted that H2-receptor antagonists such as, ranitidine, cimetidine and famotidine are able to significantly reduce the clastogenic effects of irradiation in human lymphocytes in vitro with a dose reduction factor (DRF) of 1.5–2 [23].

With regard to the reduction in the number of hematological cells as a result of irradiation therapy, apoptosis has been revealed to be the main culprit. Being a strong scavenger of ^·^OH, HOCl, and NH2Cl, H2-receptor antagonists have been confirmed to make a significant impact on reducing the number of apoptosis being induced in leukocytes due to gamma irradiation [24].

In another study antioxidation and immunomodulation have been proposed as influential mechanisms of cimetidine radioprotective effects [25]. Notwithstanding, evidence shows that famotidine does not possess an immunomedulatory role in immune system, while it has the ability of scavenging oxygen radicals [26].

One of the consequences of DNA damage and repair is micronucleus formation. The number of micronucleated cells represents the cytogenetic damage caused by X-irradiation. Cimetidine was found to have a dose-dependent protective significant effect against radiation-induced micronucleus formation in human peripheral blood lymphocytes [27].

In an experimental study to assess a combination of melatonin and famotidine as radioprotectors, Samei et al. has concluded that the radioprotective effect of combined famotidine and melatonin was the same as famotidine alone. However, famotidine has been shown to be a good radioprotective agent for normal tissue, its accretion in tumor tissues may cause a reduction in the efficacy of radiotherapy. What’s more, the results of the 2,2-diphenylpicrylhydrazyl (DPPH) assay, has demonstrated that famotidine did not have any antioxidant capacity and the 20 µg/ml and 40 µg/ml concentrations did not differ significantly. Even so, the 80 µg/ml dose of famotidine was significantly different from other doses [18].

A dose of 5 mg/kg for famotidine was suggested to be significantly effective before 4 Gy irradiation, resulting in approximately 50% reduction in DNA damage in vivo in mouse leukocytes [28].

It has been shown in another study that with regard to radioprotective role, famotidine alone can be as effective as it is in combination with other drugs [29]. An in vivo study has indicated that the radioprotection effects of oral famotidine and cimetidine alone is similar to a combination of these drugs, as the DRFs of 1.09, 1.01, and 1.08 were reported for famotidine + cimetidine, cimetidine and famotidine, respectively. Another compelling result of aforementioned study showed that increasing the concentration of these drugs had significantly led to a difference in survival fraction. In spite of being exposed to a lethal dose, 50% (LD50) radiation dose, 100% survival was witnessed using 8 mg/kg for famotidine and 40 mg/kg for cimetidine [29].

A DRF of 1.5–2 has been previously reported for cimetidine, ranitidine and famotidine. All three drugs were revealed to significantly decrease the frequency of radiation-induced micronuclei and chromosomal abnormalities at different doses. However, famotidine was shown to be significantly more effective compared to either cimetidine or ranitidine [30].

## Conclusion

Our findings proved that famotidine was an effective radioprotector in terms of the lymphocyte and platelet counts, so that at the end of the study patients in famotidine group showed significantly higher frequencies of lymphocytes and platelets. Besides, in the famotidine group, a significant reduction in thrombocytopenia was observed. To the best of the author’s knowledge, there is a small number of studies to assess famotidine as a radioprotector, most of which are in vivo. Therefore, drawing a favorable comparison between the existing evidence and the results we secured in this study is somewhat difficult. We recommend conducting further research studies and clinical trials in various cancer patient populations using different doses of famotidine or a combination of radioprotective agents to suggest an optimum dose and provide more information about the possible side effects of such drugs in different populations and conditions.

## Data Availability

The datasets used and analyzed during the current study are available from the corresponding author on reasonable request.

## Abbreviations

PPIs: Proton pump inhibitors
CBC: Complete blood count
SD: Standard deviation
MD: Mean difference
Mdn: Median
EGJ: Esophago-gastric junction
WBC: White blood cells
NMRI: Naval Medical Research Institute
MnPCEs: Micro-nucleated polychromatic erythrocytes
DRF: Dose reduction factor
DPPH: 2,2-Diphenylpicrylhydrazyl
LD50: Lethal dose, 50%
